# Cannabis use, sexual behaviors, and HIV prevention behaviors among young adults attending key-population-led sexual health clinics in Bangkok, Thailand: A mixed-method study

**DOI:** 10.1371/journal.pone.0352395

**Published:** 2026-06-26

**Authors:** Pongkwan Yimsaard, Kathryn E. Lancaster, Thunchanok Ratchatasitthigul, Rena Janamnuaysook, Kritima Samitpol, Nittaya Phanuphak, Somporn Saiwaew, Jeremy L. Ross, Arunrat Tangmunkongvorakul

**Affiliations:** 1 Department of Psychiatry, Faculty of Medicine, Chulalongkorn University, Bangkok, Thailand; 2 Department of Psychiatry, King Chulalongkorn Memorial Hospital Thai Red Cross Society, Bangkok, Thailand; 3 Department of Implementation Science, Division of Public Health Sciences, Wake Forest University School of Medicine, United States of America; 4 Institute of HIV Research and Innovation, Bangkok, Thailand; 5 Center of Excellence in Transgender Health, Chulalongkorn University, Bangkok, Thailand; 6 Rainbow Sky Association of Thailand, Bangkok, Thailand; 7 TREAT Asia/amfAR – The Foundation for AIDS Research, Bangkok, Thailand; 8 Research Institute for Health Sciences, Chiang Mai University, Thailand; Centers for Disease Control and Prevention, UNITED STATES OF AMERICA

## Abstract

**Background:**

The intersection of cannabis and sexual behaviors poses important public health concerns, particularly following Thailand’s cannabis decriminalization. However, evidence on how cannabis use relates to sexual and HIV prevention behaviors among young adults in this policy context remains limited. This mixed-methods study aimed to assess cannabis use patterns and their associations with sexual behaviors and HIV prevention behaviors among young adults attending key population-led sexual health clinics in Bangkok, Thailand.

**Methods:**

A self-report survey of 200 participants aged 18–24 assessed cannabis use, sexual behaviors, and HIV prevention behaviors using study-specific questionnaires, and the Cannabis Use Disorder Identification Test-Revised. Laboratory data, including HIV and Sexually Transmitted Infections (STIs) test results were extracted from medical records. Chi-square tests and Poisson regression examined associations between cannabis use, sexual behaviors, and HIV prevention behaviors. In-depth interviews with 15 cannabis users and 15 non-users explored perceptions of cannabis use in relation to sexual behaviors and HIV prevention behaviors to complement quantitative findings. Thematic analysis was conducted.

**Results:**

Among the 200 participants (mean age = 21.45, SD = 1.89; 35% gay men, 32% transgender women), 22% were past-month cannabis users. Cannabis use was significantly associated with sex under the influence of any substances (Prevalence Ratio (PR)=1.24, 95%CI: 1.09,1.41), and alcohol (PR = 1.12, 95%CI: 1.02,1.24). No associations were found with other sexual behaviors, PrEP adherence, HIV or any STIs test results. Many participants cited disinhibitions as a pathway to risk, whilst some cannabis users emphasized individual responsibility as a more important determinant of behavior.

**Conclusions:**

Cannabis use is linked to alcohol- or substance-influenced sex. Diverging views on cannabis and sexual risk suggest a need for tailored youth-centered harm reduction strategies within sexual health clinics that address risks and empower personal responsibility, particularly in the context of Thailand’s evolving cannabis policy.

## Introduction

Cannabis use among young adults is increasing globally. According to a U.S. nationally representative survey, past 30-day cannabis use among young adults aged 19–30 increased from 16.6% in 2012 to 28.8% in 2022 [1]. A 2023 cross-sectional survey in Thailand showed that the prevalence of past-month cannabis use was 21.2% for males and 8.7% for females aged 20–24 years, with more than half initiating use after cannabis decriminalization in 2022 [2].

Despite the increasing prevalence of cannabis use, the relationship between cannabis use and sexual behaviors or HIV pre-exposure prophylaxis (PrEP) adherence among young adults remains inconclusive. Some studies suggest that cannabis users are more likely than non-users to engage in condomless sex [3,4], have multiple sexual partners [5], engage in alcohol or substances-influenced sex [4], transactional sex [4,6], and PrEP discontinuation [7], all of which may increase their risk for HIV and other sexually transmitted infections (STIs). However, other studies have found inconsistent or no significant associations. For instance, a US cross-sectional study did not observe a link between cannabis use and condomless sex [8]. Similarly, findings from a US cohort study conduct among young men who have sex with men (MSM) found a lower incidence of STIs among cannabis users compared to those who reported no drug or other drug use [9]. Furthermore, a US study reported no significant relationship between cannabis use and PrEP adherence among gay and bisexual men [10].

Currently, there is limited research on cannabis use in Thailand, especially regarding its patterns and implications for sexual health among young adults, a key population for HIV prevention. No Thai study has yet examined the association between cannabis use, sexual behaviors, and HIV prevention behaviors. Understanding cannabis use in this population is crucial for assessing potential risks and developing targeted interventions, especially considering Thailand’s recent cannabis policy changes.

This study aims to determine the prevalence of cannabis use, examine cannabis use patterns, and assess the associations between cannabis use and sexual behaviors as well as HIV prevention behaviors among young adults attending sexual health clinics in Bangkok, Thailand. Using a mixed-methods approach allows for both quantifying these associations and gaining deeper insight into participants’ perceptions. By exploring the relationship between cannabis use, sexual behaviors and HIV prevention behaviors, it is hoped this study will provide valuable insights for HIV and other STI prevention efforts among young adults in Thailand.

## Methods

### Study design

This study employed a mixed-methods design, consisting of two distinct phases: quantitative followed by qualitative [11]. This study was approved by Chulalongkorn University Institute Review Board (COA No. 1262/2023). This manuscript was reported following the Good Reporting of a Mixed Methods Study (GRAMMS) guidelines [12].

### Setting and sample

This study was conducted at two key-population-led community sexual health clinics: The Pribta-Tangerine clinic and the Rainbow Sky Association of Thailand clinic, both located in Bangkok, Thailand. Both clinics provide comprehensive sexual health services, including HIV testing, PrEP, and STI screening and treatment, to individuals of all genders, with a focus on key populations such as MSM and transgender individuals. The target sample was 200 young adults (aged 18–24 years) attending the clinics for either HIV prophylaxis, HIV testing, or STI testing, including syphilis, gonorrhea, chlamydia, hepatitis C (HCV), and hepatitis B (HBV). Exclusion criteria included known HIV-positive status or prior treatment for HIV before enrollment, as the study focused on HIV prevention and sexual behaviors among individuals at risk, whose contexts differ from those living with HIV.

The sample size was estimated using Cochran’s formula [13], based on a 2% prevalence of cannabis use in Thailand [14], with a precision of 2%. To account for an anticipated 5% attrition, the final sample size was set at 200 participants.

For the qualitative phase, a subset of 30 participants was purposively selected from the 200 participants in the quantitative phase, consisting of 15 cannabis users and 15 non-users.

Participants were offered an information sheet and provided written consent before participating in both quantitative and qualitative phases of the study.

### Data collection and measures

#### Quantitative Phase.

A total of 200 participants were recruited using convenience sampling between November 27, 2023 to March 29, 2024. Eligible individuals were approached during clinic visits and invited to participate. Of the 220 eligible individuals approached, 200 agreed to participate resulting in a 90.9% acceptance rate. Participants completed an anonymous, self-administered survey on a tablet, and relevant laboratory data were extracted from their medical records on the same day as their survey completion. Each participant received compensation equivalent to USD 9 in local currency.

The survey took approximately 20 minutes to complete and included questions across several domains: demographic information (i.e., age, sex, gender, education, income, work status, and marital status), sexual behavior, PrEP adherence, alcohol and substance use data, and cannabis use-related data.

### Sexual behavior data and PrEP adherence

Sexual behavior data were collected using a study-specific questionnaire, where participants were asked whether they had engaged in vaginal or anal sex in the past 3 months. Those who answered “yes” were then asked to report the number of sexual partners they had for each type of sex, as well as the frequency of condom use during intercourse, with the following response options: every time, sometimes, rarely, and never. Additionally, participants were asked to rate how often, in the past 3 months, they had used any drugs or consumed alcohol before or during sex, and whether they had exchanged sex for money, using the same response options.

Participants were also asked whether they currently use PrEP. If they did, they were further asked to rate their PrEP adherence over the past month using a 6-point Likert scale, ranging from “very poor” to “excellent.”

### Alcohol, tobacco and substance use data

Participants were asked whether they had ever consumed alcohol in their lifetime. Those who reported lifetime alcohol use were then asked to complete the Alcohol Use Disorder Identification Test-Consumption (AUDIT-C). An AUDIT-C score of ≥4 was considered indicative of unhealthy alcohol use in this study [15]. For tobacco use, participants were asked whether they currently smoke, with response options of not at all, daily, or less than daily. Other substance use was assessed by asking participants whether they had ever used any of the following substances: cannabis, amphetamine-type stimulants, cocaine, laughing gas, poppers, sleeping pills, hallucinogens, heroin, ecstasy, tramadol, kratom and other substances. Participants who reported lifetime use of any substance were subsequently asked whether they had used it in the past month.

### Cannabis use data

Participants who reported past-month cannabis use were asked to complete the Cannabis Use Disorder Identification Test-Revised (CUDIT-R). The CUDIT-R is an 8-item self-report screening tool that assesses various aspects of cannabis use and related problems, including frequency, quantity, and negative consequences. The total score ranges from 0 to 32, with a score of 8 or higher indicating hazardous use and a score of 12 or higher indicating probable cannabis use disorder [16].

We also assessed whether participants have ever used cannabis simultaneously with any of the following substances: alcohol, amphetamine-type stimulants, laughing gas, poppers, sleeping pills, hallucinogens, heroin, ecstasy, tramadol, kratom and other substance. Participants responded with “yes” or “no” to each substance.

Participants were also asked to report their most frequent method of cannabis consumption in the past month, with options including inhalation (joint, blunt, bong, vaporizer, dabbing), ingestion (food, drink, capsule, CBD oil), sublingual spray, and topical application.

### Laboratory Data

Laboratory data were extracted from clinic records based on routine testing conducted as part of each clinic’s standard clinical care. For all participants who completed the survey (n = 200), we retrieved HIV and other STI test results (gonorrhea, syphilis, chlamydia, HBV, and HCV) recorded on the date of survey completion. As testing was performed according to their clinical guidelines, not all participants were tested for all conditions.

### Qualitative phase

During the qualitative phase (May 21, 2024 – September 19, 2024), we purposively selected 15 cannabis users and 15 non-users from the 200 survey respondents, resulting in a total of 30 participants. These participants were invited to take part in semi-structured interviews (SSIs) to explore perceptions of cannabis use in relation to sexual behaviors and HIV prevention behaviors. The interview guide was developed based on the study objectives and existing literature on cannabis use and sexual risk behaviors. Examples of questions used in the SSIs are provided in Supplementary Table 1.

The SSIs were conducted in private rooms at the clinics. All SSIs were conducted in Thai, audio recorded with participants’ consent, and ranged in duration from 32 to 62 minutes, with an average length of 46.07 minutes (SD = 9.16). Participants received the local currency equivalent of 24 USD as compensation for their time. Each SSI was facilitated using a structured interview guide by either the principal investigator (a psychiatrist), an MSc-level staff member, or a PhD-level staff member, all of whom were cisgender women trained in qualitative research.

### Data analysis

#### Quantitative data.

Descriptive statistics were used to summarize the demographic characteristics of the study population, including frequencies, percentages, means, and standard deviations (SD). Chi-square test or Fisher’s exact test was conducted to examine the relationships between cannabis use status (past-month user vs. non-user) and each categorical variables, including sociodemographic, sexual behavior, PrEP adherence, laboratory data and alcohol and substance data. For continuous variables, independent t-tests were used to compare means between groups.

Poisson regression was used to estimate the prevalence ratio (PR) of cannabis use, adjusting for sociodemographic and substance use variable. Only significant variables from the univariate analysis, including those related to sexual behavior data and PrEP adherence were included as independent variables in the model, with past-month cannabis use status as the dependent variable. All analyses were conducted using SPSS version 29, with a significance level set at p < 0.05.

#### Qualitative data.

All audio files were transcribed in Thai by local professional consultants. The Thai transcripts were imported into Dedoose software for qualitative data management and coding, which was conducted in Thai using a thematic analysis approach. Relevant quotations were later translated into English for reporting.

In the first step, descriptive coding was applied to systematically categorize key sexual behaviors reported in the quantitative survey, including inconsistent condom use, multiple sexual partners, transactional sex, substance-influenced sex, and PrEP adherence. Following the initial coding, a thematic analysis was performed to identify broader themes emerging inductively from the coded data, providing deeper insights into the underlying factors influencing these behaviors (Supplementary Table 1).

Two coders, both cisgender women trained in qualitative research methods, independently coded all interviews. Discrepancies in coding were reviewed and resolved through discussion to ensure consistency and reliability. The team engaged in reflexivity through regular discussion to acknowledge and minimize potential biases during analysis.

### Triangulation of data

By employing a triangulation approach, this study utilized convergence analysis to compare findings from different methods to expand understanding by examining the extent to which the quantitative and qualitative results converged or diverged. We first analyzed quantitative and qualitative results separately and then compared results. Where possible, we have an integrated presentation of results.

## Results

### Quantitative survey

#### Sample characteristics.

At the enrollment, 200 participants were recruited. The average age of participants was 21.45 years (SD = 1.79). Eighty-five percent were assigned male at birth with 35.5% identifying themselves as gay men, 32% as transgender women, and 11% as men. The majority (70%) of participants reported having completed education beyond grade 12. Additionally, 57% of participants were students and 8% reported living with long-term partners (Table 1).

**Table 1:** Sociodemographic Characteristics, Substance Use, Sexual Behaviors, HIV Prevention Behaviors, and HIV/STI Laboratory Results Among Young Adults Attending Key Population-Led Sexual Health Clinics in Bangkok, Thailand: Overall and Stratified by Past-Month Cannabis Use (N = 200).

Characteristics	Overall, N = 200		Past-month Cannabis Users, n = 44		Non-users, n = 156		X^2^	p-value
	n	%	n	%	n	%		
**Age group** 18-20 years old 21-24 years old	67 133	33.5 66.5	14 30	31.8 68.2	53 103	34.0 66.0	0.07	0.789
**Sex at birth** Female Male Prefer not to answer	24 171 5	12.0 85.5 2.5	7 37 0	15.9 84.1 0	17 134 5	10.9 85.9 3.2	–	0.372^a^
**Gender/Sexual Orientation** Man Woman Transgender woman Transgender man Gay man Gender variance/non-binary Questioning	22 30 64 2 71 9 2	11.0 15.0 32.0 1.0 35.5 4.5 1.0	6 8 13 0 13 3 1	13.6 18.2 29.5 0 29.5 6.8 2.3	16 22 51 2 58 6 1	10.3 14.1 32.7 1.3 37.2 3.8 0.6	–	0.645^a^
**Education** Grade 12 or lower Higher than grade 12	60 140	30.0 70.0	14 30	31.8 68.2	46 110	29.5 70.5	0.09	0.766
**Work status** Employed Unemployed Student	70 16 114	35.0 8.0 57.0	16 6 22	36.4 13.6 50.0	54 10 92	34.6 6.4 59	2.76	0.252
**Income (Baht)**^**b**^ Less than 15,000 15,000 and above Prefer not to answer	107 84 9	53.5 42.0 4.5	20 23 1	45.5 52.3 2.3	87 61 8	55.8 39.1 5.1	2.72	0.256
**Marital status** Single/ Separated Living with a long-term partner	184 16	92.0 8.0	39 5	88.6 11.4	145 11	92.9 7.1	–	0.353^a^
**AUDIT-C score, n = 186** Less than 4 4 and above	89 97	47.8 52.2	11 33	25.0 75.0	78 64	54.9 45.1	12.06	**<0.001**
**Tobacco usage** Not at all Daily Less than daily	178 12 10	89.0 6.0 5.0	31 6 7	70.5 13.6 15.9	147 6 3	94.2 3.8 1.9	–	**<0.001** ^**a**^
**Cannabis usage** Lifetime use *Yes* *No* Past-month use *Yes* *No*	75 125 44 156	37.5 62.5 22.0 78.0	44 0 – –	100 0 – –	31 125 – –	19.9 80.1 – –	94.02 –	**<0.001** –
**Other substance use (lifetime)** Poppers Laughing gas Kratom Amphetamine-type stimulant Sedative drug Hallucinogen Ecstasy Cocaine Heroin Tramadol Other	46 26 23 15 13 7 3 3 1 1 18	23.0 13.0 11.5 7.5 6.5 3.5 1.5 1.5 0.5 0.5 9.0	10 16 13 7 5 3 2 3 1 1 8	22.7 36.4 29.5 15.9 11.4 6.8 4.5 1.5 2.3 2.3 18.2	36 10 10 8 8 4 1 0 0 0 10	23.1 6.4 6.4 5.1 5.1 2.6 0.6 0 0 0 6.4	0.002 27.23 18.05 – – – – – – – –	0.961 **<0.001** **<0.001** **0.025**^**a**^ 0.165^a^ 0.351^a^ 0.122^a^ **0.010**^**a**^ 0.220^a^ 0.220^a^ **0.022**^**a**^
**Other substance use (past-month)** Poppers Laughing gas Kratom Amphetamine-type stimulant Sedative drug Hallucinogen Ecstasy Cocaine Heroin Tramadol Other	42 22 17 12 12 6 2 2 1 1 18	21.0 11.0 8.5 6.0 6.0 3.0 1.0 1.0 0.5 0.5 9.0	9 15 10 5 5 2 1 2 1 1 8	20.5 34.1 22.7 11.4 11.4 4.5 2.3 4.5 2.3 2.3 18.2	33 7 7 7 7 4 1 0 0 0 10	21.2 4.5 4.5 4.5 4.5 2.6 0.6 0 0 0 6.4	0.01 30.72 14.68 – – – – – – – –	0.920 **<0.001** **<0.001** 0.141^a^ 0.141^a^ 0.615^a^ 0.392^a^ **0.048**^**a**^ 0.220^a^ 0.220^a^ **0.022**^**a**^
**Number of substances used in the past month** No substance 1 substance 2 substances or more	97 61 42	48.5 30.5 21.0	0 14 30	0 31.8 68.2	97 47 12	62.2 30.1 7.7	87.19	**<0.001**
**Condom use (Vaginal sex), n = 36** Every time Sometimes/Rarely/Never	13 23	36.1 63.9	3 10	23.1 76.9	10 13	43.5 56.5	–	0.292^a^
**Condom use (Anal sex), n = 118** Every time Sometimes/Rarely/Never	55 63	46.6 53.4	10 16	38.5 61.5	45 47	48.9 51.1	0.89	0.346
**Sexual partner (Vaginal sex), n = 36** Single partner More than one partner	16 20	44.4 55.6	3 10	23.1 76.9	13 10	56.5 43.5	3.76	0.052
**Sex partner (Anal sex), n = 118** Single partner More than one partner	62 56	52.5 47.5	13 13	50.0 50.0	49 43	53.3 46.7	0.09	0.769
**Alcohol use before or during sex** Every time/sometime/rarely Never	86 114	43.0 57.0	31 13	70.5 29.5	55 101	35.3 64.7	17.35	**<0.001**
**Any drug use before or during sex** Every time/sometime/rarely Never	33 167	16.5 83.5	18 26	40.9 59.1	15 141	9.6 90.4	24.35	**<0.001**
**Exchange sex for money** Every time/sometime/rarely Never	17 183	8.5 91.5	9 35	20.5 79.5	8 148	5.1 94.9	–	**0.003** ^**a**^
**PrEP use** Current use Not current use	68 132	34.0 66.0	12 32	27.3 72.7	56 100	35.9 64.1	1.14	0.286
**Self-rate PrEP adherence, n = 68** Very poor Poor Fair Good Very good Excellent	0 2 7 20 21 18	0 2.9 10.3 29.4 30.9 26.5	0 0 1 3 3 5	0 0 8.3 25.0 25.0 41.7	0 2 6 17 18 13	0 3.6 10.7 30.4 32.1 23.2	–	0.818^a^
**HIV test at enrollment, n = 200** Positive Negative	3 197	1.5 98.5	1 43	2.3 97.7	2 154	1.3 98.7	–	0.527^a^
**VDRL, n = 185** Positive Negative	14 171	7.6 92.4	5 38	11.6 88.4	9 133	6.3 93.7	–	0.321^a^
**Chlamydia, n = 106** Positive Negative	24 82	22.6 77.4	6 21	22.2 77.8	18 61	22.8 77.2	0.004	0.952
**Gonorrhea, n = 105** Positive Negative	15 90	14.3 85.7	3 23	11.5 88.5	12 67	15.2 84.8	–	0.757^a^
**Hepatitis C, n = 114** Positive Negative	3 111	2.6 97.4	0 26	0 100	3 85	3.4 96.6	–	1.00^a^
**Hepatitis B, n = 61** Positive Negative	0 61	0 100.0	0 18	0 100	0 43	0 100	–	–
**CUDIT-R, n = 44** Low-risk user Hazardous user Possible cannabis use disorder			30 9 5	68.2 20.5 11.4				
**Route of administration, n = 44** Joint Edible product Bong Blunt Vaporizing device			17 11 8 7 1	38.6 25.0 18.2 15.9 2.3				
**Co-use with cannabis, n = 44** Alcohol Laughing gas Kratom Poppers Amphetamine-type stimulant Other			27 7 5 1 1 7	61.4 15.9 11.4 2.3 2.3 15.9				

***Legend:*** *n = number of participants; % = percentage; χ² = chi-square test statistic; PrEP = pre-exposure prophylaxis; AUDIT-C = Alcohol Use Disorders Identification Test – Consumption; CUDIT-R = Cannabis Use Disorders Identification Test – Revised; ᵃFisher’s exact test;* ^*b*^*15,000 Baht ≈ 430 USD*

### Sexual behaviors and PrEP adherence

Almost sixty percent (59%, n = 118) of participants reported having engaged in anal sex, while 18% (n = 36) reported vaginal sex in the past 3 months. Among those, more than half reported inconsistent condom use during vaginal (63.9%) and anal sex (53.4%). Approximately half of the participants reported having more than one sexual partner, including 55.6% for vaginal intercourse and 47.5% for anal intercourse. Forty-three percent of participants engaged in sexual activity while consuming alcohol, 16.5% engaged in sexual activity while using drug, and 8.5% reported exchanging sex for money (Table 1). Chi-square analysis revealed that cannabis users were significantly more likely to report alcohol-influenced sex (70.5% vs 35.3%, χ² = 17.35, p < 0.001), substance-influenced sex (40.9% vs 9.6%, χ² = 24.35, p < 0.001) and engaging sex for money (20.5% vs 5.1%, Fisher’s Exact Test, p = 0.003). Around one-third (34%) of participants were current PrEP users. More than half (57.4%) of PrEP users rated their PrEP adherence over the past month as either “Very Good” or “Excellent”.

### Alcohol, tobacco and substance use data

Most participants (93%) reported lifetime alcohol use, with 52.2% having an AUDIT-C score ≥ 4. Eleven percent were current smokers. Nearly half of the participants (48.5%) reported no substance use in the past month, while 30.5% reported using a single substance, and 21.0% reported using ≥2 substances. Cannabis was the most reported substance used in the past month (22%), followed by poppers (21%) and laughing gas (11%) (Table 1).

### Cannabis-related data

Past-month cannabis users were more likely to have an AUDIT-C score ≥4 (75% vs. 45.1%, χ² = 12.06, p < 0.001) and to report daily smoking (13.6% vs. 3.8%, Fisher’s Exact Test, p < 0.001) compared to non-users. They were also significantly more likely to report using two or more substances (excluding alcohol and smoking) in the past month (68.2% vs. 7.7%, χ² = 87.19, p < 0.001) (Table 1).

Among past-month cannabis users, 11.4% were classified as possible cases of cannabis use disorder and 20.5% as hazardous users, based on the CUDIT-R. Smoking joints was the most reported method of consumption (38.6%). Nearly two-thirds (61.4%) reported co-using alcohol.

### Laboratory Data

At enrollment, the HIV prevalence among our sample was 1.5% (n = 200). Among the participants tested, 7.6% had positive VDRL results (n = 185), 22.6% tested positive for gonorrhea (n = 106), 14.3% tested positive for Chlamydia (n = 105), and 2.6% tested positive for HCV (n = 114). There were no significant differences between cannabis users and non-users for any of these infections (Table 1).

### Qualitative interview

Thirty SSIs participants included 15 cannabis users (Mean age 21.87, SD = 1.99, 40% transgender women, 46.7% student, 93.3% single) and 15 non-cannabis users (Mean age 20.87 years, SD = 2.03, 40% transgender women, 53.3% students, 100% single) (Table 2).

**Table 2:** Sociodemographic Characteristics of Young Adults Participating in Qualitative Interviews (N = 30).

Characteristics	Overall, n (%)	Cannabis users, n (%)	Non-users, n (%)
**Age in year (Mean (SD))**	21.57 (1.87)	21.87 (1.99)	20.87 (2.03)
**Sex at birth** Female Male	4 (13.33) 26 (86.67)	3 (20) 12 (80)	1(6.7) 14 (93.3)
**Gender** Gay man Transgender woman Male Female Gender variance/non-binary	11 (36.67) 12 (40) 3 (10) 3 (10) 1 (3.33)	4 (26.7) 6 (40) 2 (13.3) 2 (13.3) 1 (6.7)	7 (46.7) 6 (40) 1 (6.7) 1 (6.7) 0 (0)
**Education** Grade 12 or lower Higher than grade 12	8 (26.67) 22 (73.33)	5 (33.3) 10 (66.7)	3 (20) 12 (80)
**Work status** Employed Unemployed Student	13 (43.33) 2 (6.67) 15 (50)	8 (53.3) 0 7 (46.7)	5 (33.3) 2 (13.3) 8 (53.3)
**Income** Less than 15,000 Baht 15,000 Baht and above Prefer not to answer	12 (40) 17 (56.67) 1 (3.33)	5 (33.3) 9 (60) 1 (6.7)	7 (46.7) 8 (53.3) 0
**Marital status** Single Living with a long-term partner	29 (96.67) 1 (3.33)	14 (93.3) 1 (6.7)	15 (100) 0
**CUDIT-R risk level** Low risk Hazardous Use Cannabis Use Disorder		10 (66.7) 3 (20) 2 (13.3)	
**Co-use** Alcohol Laughing gas Kratom Other		11 (73.3) 5 (33.3) 2 (13.3) 2 (13.3)	

***Legend:*** *n = number of participants; % = percentage; CUDIT-R= The Cannabis Use Disorder Identification Test – Revised; SD= Standard Deviation*

### Sexual Behavior and Cannabis use

Quantitative analysis revealed that cannabis use was significantly associated with engaging in sex under the influence of any substance (Prevalence Ratio (PR) = 1.24, 95% CI: 1.09–1.41) and alcohol-influenced sex (PR = 1.12, 95% CI: 1.02–1.24) (Table 3). Complementing these findings, qualitative data revealed an emerging theme of lowered inhibition, characterized by a diminished capacity for self-control that led participants to engage in situations they might otherwise avoid, such as accepting substances or engaging in unplanned sex. As one participant shared:

**Table 3:** Associations Between Past-Month Cannabis Use and Sexual Behaviors Among Young Adults Attending Key Population-Led Sexual Health Clinics in Bangkok, Thailand (N = 200): Results from Poisson Regression Analysis.

Predictor	Prevalence Ratio (PR)	95% Confidence Interval	p-value	
**Alcohol use before/during sex**				
Ever	1.12	1.02, 1.24	**0.022**	
Never	–	–		
**Drug use before/during sex**				
Ever	1.24	1.09, 1.41	**0.001**	
Never	–	–		
**Engage in Sex Exchange**				
Ever	1.15	0.98, 1.36	0.090	
Never	–	–		

***Legend:*** *Dependent variable: Cannabis use (past-month use)***,** *The category “Never” serves as the reference group. The model controlled for sociodemographic variables including age, sex, education, marital status, employment status, and income, AUDIT-C score, Smoking status and number of substance use. Significant p-values (p < 0.05) were bold.*

*“I never got cocaine myself. I always received it from my partner. Normally, if it wasn’t available, I wouldn’t even think about using it. But when he had it and offered, I would just go along with him. However, on days when I wasn’t using cannabis, there were times when I was able to refuse cocaine.” [Cannabis user-01]*

Another participant described how cannabis use contributed to spontaneous sexual encounter:

*“I didn’t use weed before going out, but I used it during my night out, like when I went to a bar at Khaosan Road, and I ended up having sex. It was totally unplanned. I didn’t intend to have sex that day.” [Cannabis user-07]*

While the quantitative data did not show a statistically significant relationship between cannabis use and inconsistent condom use, multiple sexual partners, HIV serology or other STI results (Table 1), the qualitative findings highlighted impaired judgement as an important contextual factor. Many participants described how cannabis intoxication might diminish their capacity to make safer sexual decision. One cannabis user explained:

*“Once I use cannabis, I stop paying attention to things like making sure to wear a condom or checking for anything. Using cannabis, for me, is mostly not about sitting quietly and using it. The environment plays a role too. Everything blends, and you don’t really pay attention to whether there’s a condom, lubricant, or anything.” [Cannabis user-01]*

Non-cannabis users, despite lacking direct experience with cannabis, also expressed concerns about its potential to impair judgement during sexual activity. As one participant remarked:

*“I think using cannabis can make you feel spaced out and lose control, similar to being drunk. While I’ve never used cannabis, I believe its effects could impair judgment during sexual activity, much like alcohol, which can also lead to similar outcomes.” [Non user-08]*

However, not all participants agreed. Three cannabis users insisted that cannabis use does not inherently increase sexual risk and instead emphasized individual responsibility:

*“No, not at all. I’ve had experience using cannabis before having sex. At that time, it was completely normal—like, I told him to use a condom, and he used it as usual. I think risky behaviors like forgetting to use a condom or engaging in group sex depend entirely on the individual, whether they choose to do that or not.” [Cannabis user-05]*

### PrEP Adherence and cannabis use

Although the quantitative analysis did not find a significant difference in PrEP adherence between cannabis users and non-users, qualitative findings revealed additional insights into perceived differences between users and non-users.

One emerging theme was impaired judgement, primarily described by non-users when reflecting on how cannabis might affect PrEP adherence. Several non-users perceived cannabis as a substance that could interfere with one’s ability to remember or prioritize taking PrEP. One participant stated:

*“For example, if someone consumes cannabis to the point of intoxication and needs to take PrEP at 8 p.m., they might be too impaired, causing them to forget their PrEP for that day. I believe it will happen 100% of the time.” [Non-user 01]*

Another highlighted the role of dosage:

*“I think it depends on how much a person consumes. If they use it to the point of feeling completely spaced out, it will definitely have an effect. They probably wouldn’t be conscious enough to think about what time they need to take their PrEP.” [Non-user 08]*

Others noted that cannabis might distort how individuals perceive the importance of PrEP:

*“I think if someone smokes cannabis, they might feel relaxed or in a daze, and they might think PreP is not a big deal. They may know they need to take PrEP, but they don’t feel it’s urgent. It’s not that they forget, but they just feel like it’s not that important.” [Non-user 02]*

In contrast, several cannabis users described their own ability to maintain adherence as a matter of individual responsibility. Many users highlighted that they had developed a routine that allowed them to take PrEP before using cannabis. One participant stated:

*“I think it depends on when a person takes PrEP. Since PrEP can be taken at any time of the day. For me, I take it in the evening, so it hasn’t been a problem. I usually only use cannabis when I go out at night.” [Cannabis user-01]*

## Discussion

This mixed-methods study offers timely insights into cannabis use and sexual behaviors among young adults attending sexual health clinics in Bangkok, a population at the forefront of HIV prevention efforts. The high prevalence of past-month cannabis use in our sample aligns with recent national studies of young adults [2,17]. Co-use of cannabis and alcohol was common, with many participants showing signs of problematic alcohol use, underscoring patterns of polysubstance use that may heighten sexual health risks. These findings suggest a need for integrating substance use screening and brief intervention strategies into routine sexual health services to help identify and address overlapping risks in this population.

Regarding cannabis and sexual behaviors, our quantitative results indicated that cannabis users were more likely to engage in alcohol-influenced and substance-influenced sex. These findings align with a multinational cross-sectional study involving young people from nine European cities, which similarly found that cannabis users reported more substance-influenced sex than non-users [4]. However, we did not observe a significant relationship between cannabis use and other sexual behaviors (i.e., condomless sex, multiple sexual partner and transactional sex). This aligns with a cross-sectional study of US adolescents aged 12–18 attending primary care clinics, which also found no association between cannabis use and condomless sex [8]. Moreover, a multi-site U.S. study of adolescent MSM aged 13–24 did not find an association between cannabis use and transactional sex [18]. However, some studies have indicated potential associations between cannabis use and sexual behaviors. For instance, a US online survey conducted among sexual minority men (aged 15–24 years) found that cannabis use was associated with condomless anal sex [3]. Similarly, an Australian telephone survey conducted among individuals aged 16–64 years found that daily cannabis use was associated with an increased likelihood of reporting multiple sexual partners in the previous year [5]. These variations in study outcomes may be attributed to differences in sample populations, study designs or other contextual factors, all of which can influence the observed relationship between cannabis use and sexual behaviors.

Our qualitative findings, however, revealed that participants who do not use cannabis believed cannabis could lead to risky sexual behaviors, such as condomless sex and multiple sexual partners, due to its disinhibiting effects, a view supported by previous research, which suggests that cannabis may reduce inhibitions and encourage risk-taking behaviors [17]. In contrast, cannabis users expressed a range of opinions on the relationship between cannabis use and sexual risk. Specifically, those who had used cannabis during sexual intercourse did not believe cannabis directly caused condomless sex, instead viewing sexual risk as dependent on individual responsibility. This finding aligns with other research suggesting that individuals who use substances in sexual contexts may perceive personal responsibility as a key factor in their sexual decision-making [19,20].

Our quantitative findings did not reveal a significant association between cannabis use and PrEP adherence. This is consistent with findings from two U.S.-based studies, which also found no significant relationship between cannabis use and PrEP adherence among Black MSM [21] and bisexual men [10]. However, our qualitative findings offer a more detailed perspective. Non-cannabis users expressed concerns that consuming high amounts of cannabis could impair PrEP adherence due to its intoxicating effects. This concern is supported by prior research indicating potential links between substance use and decreased adherence to HIV prevention strategies. For example, a U.S. survey among sexual minority men and gender-diverse individuals found that frequent cannabis use was associated with a higher likelihood of PrEP discontinuation [7]. This contrasts with cannabis users in our study who reported that their cannabis use did not interfere with their ability to adhere to PrEP.

The differing perspectives on cannabis and sexual behaviors between cannabis users and non-users highlight the need for tailored public health messaging. Non-users often associate cannabis uses with increased risk-taking due to its potential to lower inhibitions, reinforcing concerns about impaired judgment during sexual encounters. In contrast, many cannabis users acknowledge these effects but emphasize their ability to make responsible choices despite them. Standard risk-based messages may not resonate with youth who view their behaviors as intentional and controllable, even when using cannabis. Instead, messaging should reflect both perceived and actual risk, engaging young adults in harm reduction strategies that acknowledge their agency while offering practical guidance, particularly in the context of Thailand’s evolving cannabis policies.

This study has several limitations to be addressed. Firstly, the data was based on participants’ self-reports, which may be subject to social desirability bias. To minimize these biases, we ensured confidentiality by informing participants about the anonymity of their responses and the measures taken to protect their privacy throughout the study. Secondly, the small sample size may limit the statistical power of our findings and their generalizability to other populations. Thirdly, the temporal gap between the quantitative and qualitative phase may affected the comparability of participants’ response, limiting the integration of findings. Lastly, the qualitative findings reflect the individual perceptions and inclinations of the participants. While these narratives provide contextual depth, they represent subjective experiences that complement rather than directly validate or contradict the quantitative results.

## Conclusion

Cannabis use was common among young adults attending sexual health clinics in Bangkok and was associated with substance- or alcohol-influenced sex, though not other sexual behaviors. From a qualitative perspective, young adults held divergent views on whether cannabis use increases sexual risk, with many users emphasizing personal responsibility over intoxication effects. These findings suggest that sexual health clinics should implement tailored, youth-centered, non-stigmatizing harm reduction strategies that balance risk education with the promotion of personal responsibility, particularly in the context of Thailand’s evolving cannabis policy. Further research is needed to explore causal pathways and long-term impacts of cannabis use on HIV-related outcomes in this population.

## Supporting information

S1 Table Examples of Semi-Structured Interview Questions and Coding Framework. (DOCX)
