# Supplementary material for: Cannabis use, sexual behaviors, and HIV prevention behaviors among young adults attending key-population-led sexual health clinics in Bangkok, Thailand: A mixed-method study
Source: PLoS One. 2026 Jun 26;21(6):e0352395. doi: 10.1371/journal.pone.0352395 (PMC13309025; doi:10.1371/journal.pone.0352395)
Supplement: S1 Table — (DOCX) [file pone.0352395.s001.docx]

**Supplementary Data**

**Supplementary Table 1:** Examples of Semi-Structured Interview Questions and Coding Framework

| Example of questions | Coding framework | | |
| --- | --- | --- | --- |
|  | **Descriptive code** | **Theme** | **Description of Theme and Quotation Example** |
| - What comes to mind when you think about cannabis use? - In your opinion, do you find any risky sexual behavior associated with cannabis?   - If yes, what are they? - How can cannabis affect someone’s ability to use PrEP? - How can cannabis use can somehow affect someone’s HIV infection? - Does cannabis effect your condom use/ PrEP / PEP use? if yes, how? *(ask only cannabis users)* - Tell me about your typical day on which you might use cannabis, and when, where, and how much you use during your day.   - What may cause you to use?   - Any use of cannabis before you go out or before sexual intercourse | Inconsistent condom use | Disinhibition | - Participants described engaging in any sexual risk behaviors due to reduced self-control and increased impulsivity under the influence/intoxication of cannabis. - ***Example:*** *“As I mentioned before, I think cannabis can lower users’ inhibitions and make them more inclined to take risks.” [Non-user- 06]* |
|  | Multiple sexual partners | Impair judgment | - Participants described impaired judgment as cannabis reducing ability to assess risks and lead to make a risky behavior. - ***Example:”*** *From my experience, once I use cannabis, I stop worrying about things like making sure to wear a condom or checking for anything.”* *[Cannabis user-01]* |
|  | Any substance-influenced sex | Personal responsibility | - Participants described the decision to engage or not engage in sexual risk behaviors as a personal responsibility rather than something impaired by cannabis. - ***Example:*** *“No, not at all. I’ve had experience using cannabis before having sex……Risky behaviors like forgetting to use a condom or engaging in group sex at parties. That depends entirely on the individual, whether they choose to do that or not.” [Cannabis user-05]* |
|  | PrEP adherence |  |  |
