# Supplementary material for: Cannabis use, sexual behaviors, and HIV prevention behaviors among young adults attending key-population-led sexual health clinics in Bangkok, Thailand: A mixed-method study
Source: PLoS One. 2026 Jun 26;21(6):e0352395. doi: 10.1371/journal.pone.0352395 (PMC13309025; doi:10.1371/journal.pone.0352395)
Supplement: Attachment — Submitted filename: Response to Reviewers_April17.docx [file pone.0352395.s003.docx]

**Response to the Editor and Reviewers’ Comments**

**Journal Requirements**

| **Reviewer’s comments** | **Response from Authors** | **Revised Text** |
| --- | --- | --- |
| 1. Please ensure that your manuscript meets PLOS ONE's style requirements, including those for file naming. | We have carefully reviewed the manuscript and ensured that it complies with PLOS ONE’s style requirements, including the guidelines for file naming. | - |
| 2. We suggest you thoroughly copyedit your manuscript for language usage, spelling, and grammar. | Thank you for your suggestion. The manuscript has been thoroughly copyedited by the authors to improve language usage, spelling, and grammar. A track-changes version highlighting all edits is provided during submission. | - |
| 3. We note that the grant information you provided in the ‘Funding Information’ and ‘Financial Disclosure’ sections do not match. When you resubmit, please ensure that you provide the correct grant numbers for the awards you received for your study in the ‘Funding Information’ section. | Thank you for your comment. We have carefully reviewed and revised the ‘Funding Information’ and ‘Financial Disclosure’ sections in online submission system to ensure consistency and accuracy. The correct grant numbers have now been provided in the ‘Funding Information’ section in accordance with the journal’s requirements. | - |
| 4. Thank you for stating the following financial disclosure: This study was funded through TREAT Asia, a program of amfAR, The Foundation for AIDS Research, with support from the US National Institutes of Health’s (NIH) National Institute of Allergy and Infectious Diseases, the Eunice Kennedy Shriver National Institute of Child Health and Human Development, National Cancer Institute, National Institute of Mental Health, National Institute on Drug Abuse, the National Heart, Lung, and Blood Institute, the National Institute on Alcohol Abuse and Alcoholism, the National Institute of Diabetes and Digestive and Kidney Diseases, and the Fogarty International Center, as part of the International Epidemiology Databases to Evaluate AIDS (IeDEA; U01AI069907). This publication is the result of funding in whole or in part by the NIH. It is subject to the NIH Public Access Policy. Through acceptance of this federal funding, NIH has been given a right to make this manuscript publicly available in PubMed Central upon the Official Date of Publication, as defined by NIH. The content of this publication is solely the responsibility of the authors and does not necessarily represent the official views of any of the governments or institutions mentioned above.   Please state what role the funders took in the study. If the funders had no role, please state: "The funders had no role in study design, data collection and analysis, decision to publish, or preparation of the manuscript." If this statement is not correct you must amend it as needed. Please include this amended Role of Funder statement in your cover letter; we will change the online submission form on your behalf. | Thank you for your comment. We confirm that the funders had no role in the study design, data collection and analysis, decision to publish, or preparation of the manuscript. This statement has been included in the cover letter as requested. | *“The funders had no role in study design, data collection and analysis, decision to publish, or preparation of the manuscript.”*  **(Cover Letter)** |
| 5. In this instance it seems there may be acceptable restrictions in place that prevent the public sharing of your minimal data. However, in line with our goal of ensuring long-term data availability to all interested researchers, PLOS’ Data Policy states that authors cannot be the sole named individuals responsible for ensuring data access (<http://journals.plos.org/plosone/s/data-availability#loc-acceptable-data-sharing-methods>).  Data requests to a non-author institutional point of contact, such as a data access or ethics committee, helps guarantee long term stability and availability of data. Providing interested researchers with a durable point of contact ensures data will be accessible even if an author changes email addresses, institutions, or becomes unavailable to answer requests.  Before we proceed with your manuscript, please also provide non-author contact information (phone/email/hyperlink) for a data access committee, ethics committee, or other institutional body to which data requests may be sent. If no institutional body is available to respond to requests for your minimal data, please consider if there any institutional representatives who did not collaborate in the study, and are not listed as authors on the manuscript, who would be able to hold the data and respond to external requests for data access? If so, please provide their contact information (i.e., email address). Please also provide details on how you will ensure persistent or long-term data storage and availability. | Thank you for your comment. We acknowledge the journal’s Data Policy regarding long-term data accessibility. To address this, we have designated a non-author institutional point of contact to handle data access requests.  Data requests can be directed to the Institute of HIV Research and Innovation, via email at communications@ihri.org.  These details have been updated in the Data Availability Statement to ensure compliance with PLOS ONE’s requirements. | *“Data availablitiy: The datasets analysed during the current study are not publicly available due to the highly sensitive nature of the data. In addition, public disposition would breach compliance with the protocol approved by our ethics committee. Controlled access to the data in a secure environment is possible upon reasonable request, subject to appropriate data-sharing and confidentiality agreements. Requests should be sent to the Institute of HIV and Research Innovation (IHRI) via communications@ihri.org, which is responsible for the data and for reviewing and coordinating data access requests in accordance with ethical approvals and data protection requirements.”*  **(Online Submission System)** |
| 6. If the reviewer comments include a recommendation to cite specific previously published works, please review and evaluate these publications to determine whether they are relevant and should be cited. There is no requirement to cite these works unless the editor has indicated otherwise. | We confirm that no specific recommendations to cite additional published works were provided by the reviewers. Therefore, no additional citations have been added. | - |

**Reviewer #1**

| **Reviewer’s comments** | **Response from Authors** | **Revised Text** |
| --- | --- | --- |
| 1. **Abstract:** *Background:* add more sentence to the background section to make a point in research gaps. | Thank you for your comment. We have revised the Background section of the abstract to better highlight the research gap. | *“However, evidence on how cannabis use relates to sexual and HIV prevention behaviors among young adults in this policy context remains limited.”*  **(Abstract, Page 2, Line 23-25)** |
| 2. *Conclusions:* it is better to make the recommendation more specific based on your research results, to make it more approachable for the reader. | Thank you for your comment. We have revised the Conclusions both in abstract and main manuscript to provide more specific recommendations. | *“Conclusions: Cannabis use is linked to alcohol- or substance-influenced sex. Divergent views on cannabis and sexual risk suggest a need for tailored, youth-centered harm reduction strategies within sexual health clinics that address risks and empower personal responsibility, particularly in the context of Thailand’s evolving cannabis policy.”*  **(Abstract, Page 3, Line 44-45)**  *“These findings suggest that sexual health clinics should implement tailored, youth-centered, non-stigmatizing harm reduction strategies that balance risk education with the promotion of personal responsibility, particularly in the context of Thailand’s evolving cannabis policy.”*  **(Conclusion, Page 24-25, Line 408-411)** |
| 3. This research is well written and easy to understand, the results is important to inform the policy maker and program coordinator for HIV prevention | Thank you for your comments. We appreciate your assessment of the manuscript and its potential relevance for HIV prevention policy and programs. | - |

**Reviewer #2**

| **Reviewer’s comments** | **Response from Authors** | **Revised Text** |
| --- | --- | --- |
| 1. I have reviewed the manuscript titled "Cannabis Use, Sexual Behaviors, and HIV Prevention Behaviors Among Young Adults Attending Key Population-Led Sexual Health Clinics in Bangkok, Thailand: A Mixed Method Study" and while I believe it is the result of interesting research, there are corrections that should be made before publication and dissemination to the community. | We thank the reviewer for their constructive feedback and for acknowledging the importance of our research. We have carefully addressed all comments and made corresponding revisions throughout the manuscript to improve its clarity and quality. | - |
| 2. I will begin with the results. The tables are not standardized; each lacks a suitable title. I suggest adding a title that clearly states the nature of the results presented, including the population and sample size in parentheses using a capital N. This will make the tables more self-explanatory. | We thank the reviewer for this suggestion. All table titles have been revised throughout the manuscript to include a clear description of the study population and the total sample size in parentheses. | *“Table 1: Sociodemographic Characteristics, Substance Use, Sexual Behaviors, HIV Prevention Behaviors, and HIV/STI Laboratory Results Among Young Adults Attending Key Population-Led Sexual Health Clinics in Bangkok, Thailand: Overall and Stratified by Past-Month Cannabis Use (N=200)”*  **(Page 10, Line 211-215)**  *“Table 2: Sociodemographic Characteristics of Young Adults Participating in Qualitative Interviews (N=30)”*  **(Page 16, Line 259-260)**  *“Table 3. Associations Between Past-Month Cannabis Use and Sexual Behaviors Among Young Adults Attending Key Population-Led Sexual Health Clinics in Bangkok, Thailand (N=200): Results from Poisson Regression Analysis”*  **(Page 21, Line 326-328)** |
| 3. The hyphens accompanying each indicator below the variables in the tables should be removed; they are unnecessary. | Thank you for your comment. All hyphens in Table 1 and Table 2 have now been removed. | - |
| 4. Lowercase n should also be used for sample headings. | We appreciate this correction. We have updated all table headers to ensure that lowercase n is used for sub-sample headings (e.g., Past-month Cannabis Users, n=44; Non-users, n=156). | *“Past-month Cannabis Users, n=44; Non-users, n=156”*  **(Table 1, page 10)** |
| 5. For Table 1, it is better to include a subheading titled "Cannables Usage" and below it, the indicators "Yes" and "No." | We thank the reviewer for this suggestion. We have restructured Table 1 to include "Cannabis Usage" section that reports both lifetime and past-month usage with explicit "Yes" and "No" indicators. To enhance clarity and avoid redundancy, cannabis has been separated from the broader substance lists; the remaining items are now categorized under "Other substance use (lifetime)" and "Other substance use (past-month). | **(Table 1, page 11-12)** |
| 6. For the income section, a legend should be added stating the equivalent in dollars. | Thank you for the suggestion. We have revised Table 1 to improve clarity for readers by adding the currency unit (Baht) to the variable header and providing an approximate USD conversion (15,000 Baht ≈ 430 USD) in Table 1 legend. | *“^b^15,000 Baht ≈ 430 USD”*  **(Table 1 legend, page 14)** |
| 7. Note that several words in the table contain plurals where they should not be used. | Thank you for your comments. We have revised all plurals word in Table 1 and Table 2. | *Men’ to ‘Man’, ‘Women’ to ‘Woman’, ‘More than one partners’ to ‘More than one partner’, ‘Joints’ to ‘Joint’, ‘Edible products’ to ‘Edible product’, ‘Bongs’ to ‘Bong’, ‘Blunts’ to ‘Blunt’*  **(Table 1, page 10-14)**  *‘Gay men’ to ‘Gay man’, ‘Transgender women’ to ‘Transgender woman’*  **(Table 2, page 16-17)** |
| 8. The percentage symbol should be removed from the results tables, as the headings already indicate that they are percentages. | Thank you for your comment. All percentage symbol in Table 1 (Page 10-14) and Table 2 (Page16-17) have now been removed. | - |
| 9. In Table 3, it is not necessary to repeat the word "reference" three times; it can be placed in the legend. | Thank you for this suggestion. We have removed the word "Reference" from the table body and added a statement to the Table 3 legend specifying that "Never" serves as the reference group. | *“The category “Never” serves as the reference group.”*  **(Table 3, page 21)** |
| 10. Table 1 is mentioned too many times in the results section; I believe once is sufficient. | Thank you for this observation. We have revised the Results section to ensure that Table 1 is cited only once at the beginning of the relevant paragraph to provide immediate context for the data. Subsequent references to Table 1 have been removed. | **(Results section, page 10-20)** |
| 11. Regarding the results and conclusions, it is suggested that the difference between the quantitative and qualitative components be explored in greater depth. While they are used in a complementary way, it is important to mention that the latter represents individual perceptions that explain the inclinations of the interviewee and does not necessarily contradict or support the findings of the quantitative section. | Thank you for this insightful comment. We have addressed the relationship between the quantitative and qualitative components by revising the Limitations section to explicitly state that the qualitative findings represent subjective experiences that complement, rather than directly validate, the quantitative results.  Additionally, we have updated the Conclusion to show how these individual perceptions (e.g., the emphasis on personal responsibility) provide necessary context for the statistical findings. | *“Lastly, the qualitative findings reflect the individual perceptions and inclinations of the participants. While these narratives provide contextual depth, they represent subjective experiences that complement rather than directly validate or contradict the quantitative results.”*  **(Limitations, Page 24, Line 399-402)**  *“From a qualitative perspective, young adults held divergent views on whether cannabis use increases sexual risk, with many users emphasizing personal responsibility over intoxication effects.”*  **(Conclusion, Page 24, Line 406-407)** |
