# Supplementary material for: Cannabis use, sexual behaviors, and HIV prevention behaviors among young adults attending key-population-led sexual health clinics in Bangkok, Thailand: A mixed-method study
Source: PLoS One. 2026 Jun 26;21(6):e0352395. doi: 10.1371/journal.pone.0352395 (PMC13309025; doi:10.1371/journal.pone.0352395)
Supplement: Peer review history

# Peer review: Decision Letter 0

**DOI:** 10.1371/journal.pone.0352395.r001

**Authors:** Yury Khudyakov

26 Mar 2026

PONE-D-25-49723

Cannabis Use, Sexual Behaviors, and HIV Prevention Behaviors Among Young Adults Attending Key-Population-Led Sexual Health Clinics in Bangkok, Thailand: A Mixed-Method Study

PLOS One

Dear Dr. Yimsaard,

Thank you for submitting your manuscript to PLOS ONE. After careful consideration, we feel that it has merit but does not fully meet PLOS ONE’s publication criteria as it currently stands. Therefore, we invite you to submit a revised version of the manuscript that addresses the points raised during the review process.

==============================

Your manuscript was reviewed by two experts in the field. Both identified important problems in your submission. Please reconsider the attached comments and provide point-by-point responses.

==============================

Please submit your revised manuscript by May 10 2026 11:59PM. If you will need more time than this to complete your revisions, please reply to this message or contact the journal office at plosone@plos.org. When you're ready to submit your revision, log on to https://www.editorialmanager.com/pone/ and select the 'Submissions Needing Revision' folder to locate your manuscript file.

Please include the following items when submitting your revised manuscript:

- A letter that responds to each point raised by the academic editor and reviewer(s). You should upload this letter as a separate file labeled 'Response to Reviewers'.
- A marked-up copy of your manuscript that highlights changes made to the original version. You should upload this as a separate file labeled 'Revised Manuscript with Track Changes'.
- An unmarked version of your revised paper without tracked changes. You should upload this as a separate file labeled 'Manuscript'.

If you would like to make changes to your financial disclosure, please include your updated statement in your cover letter. Guidelines for resubmitting your figure files are available below the reviewer comments at the end of this letter.

If applicable, we recommend that you deposit your laboratory protocols in protocols.io to enhance the reproducibility of your results. Protocols.io assigns your protocol its own identifier (DOI) so that it can be cited independently in the future. For instructions see: https://journals.plos.org/plosone/s/submission-guidelines#loc-laboratory-protocols. Additionally, PLOS ONE offers an option for publishing peer-reviewed Lab Protocol articles, which describe protocols hosted on protocols.io. Read more information on sharing protocols at https://plos.org/protocols?utm_medium=editorial-email&utm_source=authorletters&utm_campaign=protocols.

We look forward to receiving your revised manuscript.

Kind regards,

Yury E Khudyakov, PhD

Academic Editor

PLOS One

Journal Requirements:

When submitting your revision, we need you to address these additional requirements.

1. Please ensure that your manuscript meets PLOS ONE's style requirements, including those for file naming. The PLOS ONE style templates can be found at

https://journals.plos.org/plosone/s/file?id=wjVg/PLOSOne_formatting_sample_main_body.pdf and

https://journals.plos.org/plosone/s/file?id=ba62/PLOSOne_formatting_sample_title_authors_affiliations.pdf

2. We suggest you thoroughly copyedit your manuscript for language usage, spelling, and grammar. If you do not know anyone who can help you do this, you may wish to consider employing a professional scientific editing service.

The American Journal Experts (AJE) (https://www.aje.com/) is one such service that has extensive experience helping authors meet PLOS guidelines and can provide language editing, translation, manuscript formatting, and figure formatting to ensure your manuscript meets our submission guidelines. Please note that having the manuscript copyedited by AJE or any other editing services does not guarantee selection for peer review or acceptance for publication.

Upon resubmission, please provide the following:

The name of the colleague or the details of the professional service that edited your manuscript

A copy of your manuscript showing your changes by either highlighting them or using track changes (uploaded as a *supporting information* file)

A clean copy of the edited manuscript (uploaded as the new *manuscript* file)

3. We note that the grant information you provided in the ‘Funding Information’ and ‘Financial Disclosure’ sections do not match.

When you resubmit, please ensure that you provide the correct grant numbers for the awards you received for your study in the ‘Funding Information’ section.

4. Thank you for stating the following financial disclosure:

This study was funded through TREAT Asia, a program of amfAR, The Foundation for AIDS Research, with support from the US National Institutes of Health’s (NIH) National Institute of Allergy and Infectious Diseases, the Eunice Kennedy Shriver National Institute of Child Health and Human Development, National Cancer Institute, National Institute of Mental Health, National Institute on Drug Abuse, the National Heart, Lung, and Blood Institute, the National Institute on Alcohol Abuse and Alcoholism, the National Institute of Diabetes and Digestive and Kidney Diseases, and the Fogarty International Center, as part of the International Epidemiology Databases to Evaluate AIDS (IeDEA; U01AI069907). This publication is the result of funding in whole or in part by the NIH. It is subject to the NIH Public Access Policy. Through acceptance of this federal funding, NIH has been given a right to make this manuscript publicly available in PubMed Central upon the Official Date of Publication, as defined by NIH. The content of this publication is solely the responsibility of the authors and does not necessarily represent the official views of any of the governments or institutions mentioned above.

Please state what role the funders took in the study. If the funders had no role, please state: "The funders had no role in study design, data collection and analysis, decision to publish, or preparation of the manuscript."

If this statement is not correct you must amend it as needed.

Please include this amended Role of Funder statement in your cover letter; we will change the online submission form on your behalf.

5. In this instance it seems there may be acceptable restrictions in place that prevent the public sharing of your minimal data. However, in line with our goal of ensuring long-term data availability to all interested researchers, PLOS’ Data Policy states that authors cannot be the sole named individuals responsible for ensuring data access (http://journals.plos.org/plosone/s/data-availability#loc-acceptable-data-sharing-methods).

Data requests to a non-author institutional point of contact, such as a data access or ethics committee, helps guarantee long term stability and availability of data. Providing interested researchers with a durable point of contact ensures data will be accessible even if an author changes email addresses, institutions, or becomes unavailable to answer requests.

Before we proceed with your manuscript, please also provide non-author contact information (phone/email/hyperlink) for a data access committee, ethics committee, or other institutional body to which data requests may be sent. If no institutional body is available to respond to requests for your minimal data, please consider if there any institutional representatives who did not collaborate in the study, and are not listed as authors on the manuscript, who would be able to hold the data and respond to external requests for data access? If so, please provide their contact information (i.e., email address). Please also provide details on how you will ensure persistent or long-term data storage and availability.

6. If the reviewer comments include a recommendation to cite specific previously published works, please review and evaluate these publications to determine whether they are relevant and should be cited. There is no requirement to cite these works unless the editor has indicated otherwise.

[Note: HTML markup is below. Please do not edit.]

Reviewers' comments:

Reviewer's Responses to Questions

**Comments to the Author**

1. Is the manuscript technically sound, and do the data support the conclusions?

The manuscript must describe a technically sound piece of scientific research with data that supports the conclusions. Experiments must have been conducted rigorously, with appropriate controls, replication, and sample sizes. The conclusions must be drawn appropriately based on the data presented.

Reviewer #1: Yes

Reviewer #2: Yes

**********

2. Has the statistical analysis been performed appropriately and rigorously?

Reviewer #1: Yes

Reviewer #2: Yes

**********

3. Have the authors made all data underlying the findings in their manuscript fully available?

The PLOS Data policy requires authors to make all data underlying the findings described in their manuscript fully available without restriction, with rare exception (please refer to the Data Availability Statement in the manuscript PDF file). The data should be provided as part of the manuscript or its supporting information, or deposited to a public repository. For example, in addition to summary statistics, the data points behind means, medians and variance measures should be available. If there are restrictions on publicly sharing data—e.g. participant privacy or use of data from a third party—those must be specified.

Reviewer #1: Yes

Reviewer #2: Yes

**********

4. Is the manuscript presented in an intelligible fashion and written in standard English?

PLOS ONE does not copyedit accepted manuscripts, so the language in submitted articles must be clear, correct, and unambiguous. Any typographical or grammatical errors should be corrected at revision, so please note any specific errors here.

Reviewer #1: Yes

Reviewer #2: Yes

**********

5. Review Comments to the Author

Please use the space provided to explain your answers to the questions above. You may also include additional comments for the author, including concerns about dual publication, research ethics, or publication ethics. (Please upload your review as an attachment if it exceeds 20,000 characters)

Reviewer #1: Abstract:

Background: add more sentence to the background section to make a point in research gaps.

Conclusions: it is better to make the recommendation more specific based on your research results, to make it more approachable for the reader.

This research is well written and easy to understand, the results is important to inform the policy maker and program coordinator for HIV prevention.

Reviewer #2: I have reviewed the manuscript titled "Cannabis Use, Sexual Behaviors, and HIV Prevention Behaviors Among Young Adults

Attending Key-Population-Led Sexual Health Clinics in Bangkok, Thailand: A MixedMethod Study" and while I believe it is the result of interesting research, there are corrections that should be made before publication and dissemination to the community.

I will begin with the results. The tables are not standardized; each lacks a suitable title. I suggest adding a title that clearly states the nature of the results presented, including the population and sample size in parentheses using a capital N. This will make the tables more self-explanatory.

The hyphens accompanying each indicator below the variables in the tables should be removed; they are unnecessary. Lowercase n should also be used for sample headings. For Table 1, it is better to include a subheading titled "Cannables Usage" and below it, the indicators "Yes" and "No." For the income section, a legend should be added stating the equivalent in dollars. Note that several words in the table contain plurals where they should not be used. The percentage symbol should be removed from the results tables, as the headings already indicate that they are percentages. In Table 3, it is not necessary to repeat the word "reference" three times; it can be placed in the legend.

Table 1 is mentioned too many times in the results section; I believe once is sufficient.

Regarding the results and conclusions, it is suggested that the difference between the quantitative and qualitative components be explored in greater depth. While they are used in a complementary way, it is important to mention that the latter represents individual perceptions that explain the inclinations of the interviewee and does not necessarily contradict or support the findings of the quantitative section.

**********

6. PLOS authors have the option to publish the peer review history of their article (what does this mean?). If published, this will include your full peer review and any attached files.

If you choose “no”, your identity will remain anonymous but your review may still be made public.

**Do you want your identity to be public for this peer review?**  For information about this choice, including consent withdrawal, please see our Privacy Policy.

Reviewer #1: No

Reviewer #2: No

**********

[NOTE: If reviewer comments were submitted as an attachment file, they will be attached to this email and accessible via the submission site. Please log into your account, locate the manuscript record, and check for the action link "View Attachments". If this link does not appear, there are no attachment files.]

To ensure your figures meet our technical requirements, please review our figure guidelines: https://journals.plos.org/plosone/s/figures

You may also use PLOS’s free figure tool, NAAS, to help you prepare publication quality figures: https://journals.plos.org/plosone/s/figures#loc-tools-for-figure-preparation.

NAAS will assess whether your figures meet our technical requirements by comparing each figure against our figure specifications.

# Author comment: Author response to Decision Letter 1

**DOI:** 10.1371/journal.pone.0352395.r002

20 Apr 2026

Dear Editor and Reviewers,

Thank you for your constructive feedback. We have provided a comprehensive, point-by-point response to all comments. For ease of reading and to preserve the formatting of our detailed responses, we have uploaded a formal 'Response to Reviewers' document. A copy of that response is also provided below per the system requirements.

Best Regards,

Pongkwan

-----------------------------------------------------------------------------------------------

Journal Requirements

1. Please ensure that your manuscript meets PLOS ONE's style requirements, including those for file naming.

• Response: We have carefully reviewed the manuscript and ensured that it complies with PLOS ONE’s style requirements, including the guidelines for file naming.

2. We suggest you thoroughly copyedit your manuscript for language usage, spelling, and grammar.

• Response: Thank you for your suggestion. The manuscript has been thoroughly copyedited by the authors to improve language usage, spelling, and grammar. A track-changes version highlighting all edits is provided during submission.

3. We note that the grant information you provided in the ‘Funding Information’ and ‘Financial Disclosure’ sections do not match. When you resubmit, please ensure that you provide the correct grant numbers for the awards you received for your study in the ‘Funding Information’ section.

• Response: Thank you for your comment. We have carefully reviewed and revised the ‘Funding Information’ and ‘Financial Disclosure’ sections in the online submission system to ensure consistency and accuracy. The correct grant numbers have now been provided in the ‘Funding Information’ section in accordance with the journal’s requirements.

4. Thank you for stating the following financial disclosure:

This study was funded through TREAT Asia, a program of amfAR, The Foundation for AIDS Research, with support from the US National Institutes of Health’s (NIH) National Institute of Allergy and Infectious Diseases, the Eunice Kennedy Shriver National Institute of Child Health and Human Development, National Cancer Institute, National Institute of Mental Health, National Institute on Drug Abuse, the National Heart, Lung, and Blood Institute, the National Institute on Alcohol Abuse and Alcoholism, the National Institute of Diabetes and Digestive and Kidney Diseases, and the Fogarty International Center, as part of the International Epidemiology Databases to Evaluate AIDS (IeDEA; U01AI069907). This publication is the result of funding in whole or in part by the NIH. It is subject to the NIH Public Access Policy. Through acceptance of this federal funding, NIH has been given a right to make this manuscript publicly available in PubMed Central upon the Official Date of Publication, as defined by NIH. The content of this publication is solely the responsibility of the authors and does not necessarily represent the official views of any of the governments or institutions mentioned above.

Please state what role the funders took in the study. If the funders had no role, please state: "The funders had no role in study design, data collection and analysis, decision to publish, or preparation of the manuscript."

If this statement is not correct you must amend it as needed.

Please include this amended Role of Funder statement in your cover letter; we will change the online submission form on your behalf.

• Response: Thank you for your comment. We confirm that the funders had no role in the study design, data collection and analysis, decision to publish, or preparation of the manuscript.

• Revised Text: This statement has been included in the cover letter as requested: “The funders had no role in study design, data collection and analysis, decision to publish, or preparation of the manuscript.”

5. In this instance it seems there may be acceptable restrictions in place that prevent the public sharing of your minimal data. However, in line with our goal of ensuring long-term data availability to all interested researchers, PLOS’ Data Policy states that authors cannot be the sole named individuals responsible for ensuring data access (http://journals.plos.org/plosone/s/data-availability#loc-acceptable-data-sharing-methods).

Data requests to a non-author institutional point of contact, such as a data access or ethics committee, helps guarantee long term stability and availability of data. Providing interested researchers with a durable point of contact ensures data will be accessible even if an author changes email addresses, institutions, or becomes unavailable to answer requests.

Before we proceed with your manuscript, please also provide non-author contact information (phone/email/hyperlink) for a data access committee, ethics committee, or other institutional body to which data requests may be sent. If no institutional body is available to respond to requests for your minimal data, please consider if there any institutional representatives who did not collaborate in the study, and are not listed as authors on the manuscript, who would be able to hold the data and respond to external requests for data access? If so, please provide their contact information (i.e., email address). Please also provide details on how you will ensure persistent or long-term data storage and availability.

• Response: Thank you for your comment. We acknowledge the journal’s Data Policy regarding long-term data accessibility. To address this, we have designated a non-author institutional point of contact to handle data access requests. Data requests can be directed to the Institute of HIV Research and Innovation, via email at communications@ihri.org.

These details have been updated in the Data Availability Statement to ensure compliance with PLOS ONE’s requirements.

• Revised Text (Online Submission): "The datasets analysed during the current study are not publicly available due to the highly sensitive nature of the data. In addition, public disposition would breach compliance with the protocol approved by our ethics committee. Controlled access to the data in a secure environment is possible upon reasonable request, subject to appropriate data-sharing and confidentiality agreements. Requests should be sent to the Institute of HIV Research and Innovation (IHRI) via communications@ihri.org, which is responsible for the data and for reviewing and coordinating data access requests in accordance with ethical approvals and data protection requirements."

6. If the reviewer comments include a recommendation to cite specific previously published works, please review and evaluate these publications to determine whether they are relevant and should be cited. There is no requirement to cite these works unless the editor has indicated otherwise.

• Response: We confirm that no specific recommendations to cite additional published works were provided by the reviewers. Therefore, no additional citations have been added.

----------------------------------------------------------------------------------------------------

Reviewer 1

1. Abstract Background: add more sentence to make a point in research gaps.

• Response: Thank you for your comment. We have revised the Background section of the abstract to better highlight the research gap.

• Revised Text: “However, evidence on how cannabis use relates to sexual and HIV prevention behaviors among young adults in this policy context remains limited.” (Abstract, Page 2, Line 23-25).

2. Conclusions: It is better to make the recommendation more specific based on your research results, to make it more approachable for the reader.

• Response: Thank you for your comment. We have revised the Conclusions in both the abstract and the main manuscript to provide more specific recommendations.

• Revised Text (Abstract): “Conclusions: Cannabis use is linked to alcohol- or substance-influenced sex. Divergent views on cannabis and sexual risk suggest a need for tailored, youth-centered harm reduction strategies within sexual health clinics that address risks and empower personal responsibility, particularly in the context of Thailand’s evolving cannabis policy.” (Abstract, Page 3, Line 44-45).

• Revised Text (Main Text): “These findings suggest that sexual health clinics should implement tailored, youth-centered, non-stigmatizing harm reduction strategies that balance risk education with the promotion of personal responsibility, particularly in the context of Thailand’s evolving cannabis policy.” (Conclusion, Page 24-25, Line 408-411).

3. This research is well written and easy to understand, the results is important to inform the policy maker and program coordinator for HIV prevention.

• Response: Thank you for your comments. We appreciate your assessment of the manuscript and its potential relevance for HIV prevention policy and programs.

----------------------------------------------------------------------------------------------------------

Reviewer 2

1. I have reviewed the manuscript titled "Cannabis Use, Sexual Behaviors, and HIV Prevention Behaviors Among Young Adults Attending Key Population-Led Sexual Health Clinics in Bangkok, Thailand: A Mixed Method Study" and while I believe it is the result of interesting research, there are corrections that should be made before publication and dissemination to the community.

• Response: We thank the reviewer for their constructive feedback and for acknowledging the importance of our research. We have carefully addressed all comments and made corresponding revisions throughout the manuscript to improve its clarity and quality.

2. I will begin with the results. The tables are not standardized; each lacks a suitable title. I suggest adding a title that clearly states the nature of the results presented, including the population and sample size in parentheses using a capital N. This will make the tables more self-explanatory.

• Response: We thank the reviewer for this suggestion. All table titles have been revised throughout the manuscript to include a clear description of the study population and the total sample size in parentheses.

• Revised Text:

- “Table 1: Sociodemographic Characteristics, Substance Use, Sexual Behaviors, HIV Prevention Behaviors, and HIV/STI Laboratory Results Among Young Adults Attending Key Population-Led Sexual Health Clinics in Bangkok, Thailand: Overall and Stratified by Past-Month Cannabis Use (N=200)” (Page 10, Line 211-215)

- “Table 2: Sociodemographic Characteristics of Young Adults Participating in Qualitative Interviews (N=30)” (Page 16, Line 259-260)

- “Table 3. Associations Between Past-Month Cannabis Use and Sexual Behaviors Among Young Adults Attending Key Population-Led Sexual Health Clinics in Bangkok, Thailand (N=200): Results from Poisson Regression Analysis” (Page 21, Line 326-328)

3. The hyphens accompanying each indicator below the variables in the tables should be removed; they are unnecessary.

• Response: Thank you for your comment. All hyphens in Table 1 and Table 2 have now been removed.

4. Lowercase n should also be used for sample headings.

• Response: We appreciate this correction. We have updated all table headers to ensure that lowercase n is used for sub-sample headings (e.g., Past-month Cannabis Users, n=44; Non-users, n=156).

• Revised Text: “Past-month Cannabis Users, n=44; Non-users, n=156” (Table 1, page 10)

5. For Table 1, it is better to include a subheading titled "Cannables Usage" and below it, the indicators "Yes" and "No."

• Response: We thank the reviewer for this suggestion. We have restructured Table 1 to include "Cannabis Usage" section that reports both lifetime and past-month usage with explicit "Yes" and "No" indicators. To enhance clarity and avoid redundancy, cannabis has been separated from the broader substance lists; the remaining items are now categorized under "Other substance use (lifetime)" and "Other substance use (past-month).

• Revised Text: see Table 1, page 11-12

6. For the income section, a legend should be added stating the equivalent in dollars.

• Response: Thank you for the suggestion. We have revised Table 1 to improve clarity for readers by adding the currency unit (Baht) to the variable header and providing an approximate USD conversion (15,000 Baht ≈ 430 USD) in Table 1 legend.

• Revised Text: “b15,000 Baht ≈ 430 USD” (Table 1 legend, page 14)

7. Note that several words in the table contain plurals where they should not be used.

• Response: Thank you for your comments. We have revised all plurals word in Table 1 and Table 2.

• Revised Text:

- Men’ to ‘Man’, ‘Women’ to ‘Woman’, ‘More than one partners’ to ‘More than one partner’, ‘Joints’ to ‘Joint’, ‘Edible products’ to ‘Edible product’, ‘Bongs’ to ‘Bong’, ‘Blunts’ to ‘Blunt’ (Table 1, page 10-14)

- ‘Gay men’ to ‘Gay man’, ‘Transgender women’ to ‘Transgender woman’ (Table 2, page 16-17)

8. The percentage symbol should be removed from the results tables, as the headings already indicate that they are percentages.

• Response: Thank you for your comment. All percentage symbols in Table 1 (Page 10-14) and Table 2 (Page 16-17) have now been removed.

9. In Table 3, it is not necessary to repeat the word "reference" three times; it can be placed in the legend.

• Response: Thank you for this suggestion. We have removed the word "Reference" from the table body and added a statement to the Table 3 legend specifying that "Never" serves as the reference group.

• Revise Text: “The category “Never” serves as the reference group.” (Table 3, page 21)

10. Table 1 is mentioned too many times in the results section; I believe once is sufficient.

• Response: Thank you for this observation. We have revised the Results section to ensure that Table 1 is cited only once at the beginning of the relevant paragraph to provide immediate context for the data. Subsequent references to Table 1 have been removed.

11. Regarding the results and conclusions, it is suggested that the difference between the quantitative and qualitative components be explored in greater depth. While they are used in a complementary way, it is important to mention that the latter represents individual perceptions that explain the inclinations of the interviewee and does not necessarily contradict or support the findings of the quantitative section.

• Response: Thank you for this insightful comment. We have addressed the relationship between the quantitative and qualitative components by revising the Limitations section to explicitly state that the qualitative findings represent subjective experiences that complement, rather than directly validate, the quantitative results. Additionally, we have updated the Conclusion to show how these individual perceptions (e.g., the emphasis on personal responsibility) provide necessary context for the statistical findings.

• Revise Text:

- “Lastly, the qualitative findings reflect the individual perceptions and inclinations of the participants. While these narratives provide contextual depth, they represent subjective experiences that complement rather than directly validate or contradict the quantitative results.” (Limitations, Page 24, Line 399-402)

- “From a qualitative perspective, young adults held divergent views on whether cannabis use increases sexual risk, with many users emphasizing personal responsibility over intoxication effects.” (Conclusion, Page 24, Line 406-407)

Attachment Submitted filename: Response to Reviewers_April17.docx

# Peer review: Decision Letter 1

**DOI:** 10.1371/journal.pone.0352395.r003

**Authors:** Yury Khudyakov, Yury Khudyakov

9 Jun 2026

Cannabis Use, Sexual Behaviors, and HIV Prevention Behaviors Among Young Adults Attending Key-Population-Led Sexual Health Clinics in Bangkok, Thailand: A Mixed-Method Study

PONE-D-25-49723R1

Dear Dr. Yimsaard,

We’re pleased to inform you that your manuscript has been judged scientifically suitable for publication and will be formally accepted for publication once it meets all outstanding technical requirements.

Within one week, you’ll receive an e-mail detailing the required amendments. When these have been addressed, you’ll receive a formal acceptance letter and your manuscript will be scheduled for publication.

An invoice will be generated when your article is formally accepted. Please note, if your institution has a publishing partnership with PLOS and your article meets the relevant criteria, all or part of your publication costs will be covered. Please make sure your user information is up-to-date by logging into Editorial Manager at Editorial Manager® and clicking the ‘Update My Information' link at the top of the page. For questions related to billing, please contact billing support.

If your institution or institutions have a press office, please notify them about your upcoming paper to help maximize its impact. If they’ll be preparing press materials, please inform our press team as soon as possible -- no later than 48 hours after receiving the formal acceptance. Your manuscript will remain under strict press embargo until 2 pm Eastern Time on the date of publication. For more information, please contact onepress@plos.org.

Kind regards,

Yury E Khudyakov, PhD

Academic Editor

PLOS One

Additional Editor Comments (optional):

Reviewers' comments:

Reviewer's Responses to Questions

**Comments to the Author**

1. If the authors have adequately addressed your comments raised in a previous round of review and you feel that this manuscript is now acceptable for publication, you may indicate that here to bypass the “Comments to the Author” section, enter your conflict of interest statement in the “Confidential to Editor” section, and submit your "Accept" recommendation.

Reviewer #1: All comments have been addressed

**********

2. Is the manuscript technically sound, and do the data support the conclusions?

The manuscript must describe a technically sound piece of scientific research with data that supports the conclusions. Experiments must have been conducted rigorously, with appropriate controls, replication, and sample sizes. The conclusions must be drawn appropriately based on the data presented.

Reviewer #1: (No Response)

**********

3. Has the statistical analysis been performed appropriately and rigorously?

Reviewer #1: (No Response)

**********

4. Have the authors made all data underlying the findings in their manuscript fully available?

The PLOS Data policy requires authors to make all data underlying the findings described in their manuscript fully available without restriction, with rare exception (please refer to the Data Availability Statement in the manuscript PDF file). The data should be provided as part of the manuscript or its supporting information, or deposited to a public repository. For example, in addition to summary statistics, the data points behind means, medians and variance measures should be available. If there are restrictions on publicly sharing data—e.g. participant privacy or use of data from a third party—those must be specified.

Reviewer #1: (No Response)

**********

5. Is the manuscript presented in an intelligible fashion and written in standard English?

PLOS ONE does not copyedit accepted manuscripts, so the language in submitted articles must be clear, correct, and unambiguous. Any typographical or grammatical errors should be corrected at revision, so please note any specific errors here.

Reviewer #1: (No Response)

**********

6. Review Comments to the Author

Please use the space provided to explain your answers to the questions above. You may also include additional comments for the author, including concerns about dual publication, research ethics, or publication ethics. (Please upload your review as an attachment if it exceeds 20,000 characters)

Reviewer #1: (No Response)

**********

7. PLOS authors have the option to publish the peer review history of their article (what does this mean?). If published, this will include your full peer review and any attached files.

If you choose “no”, your identity will remain anonymous but your review may still be made public.

**Do you want your identity to be public for this peer review?**  For information about this choice, including consent withdrawal, please see our Privacy Policy.

Reviewer #1: No

**********

# Editor report: Acceptance letter

**DOI:** 10.1371/journal.pone.0352395.r004

**Authors:** Yury Khudyakov, Yury Khudyakov

PONE-D-25-49723R1

PLOS One

Dear Dr. Yimsaard,

I'm pleased to inform you that your manuscript has been deemed suitable for publication in PLOS One. Congratulations! Your manuscript is now being handed over to our production team.

At this stage, our production department will prepare your paper for publication. This includes ensuring the following:

* All references, tables, and figures are properly cited

* All relevant supporting information is included in the manuscript submission,

* There are no issues that prevent the paper from being properly typeset

You will receive further instructions from the production team, including instructions on how to review your proof when it is ready. Please keep in mind that we are working through a large volume of accepted articles, so please give us a few days to review your paper and let you know the next and final steps.

Lastly, if your institution or institutions have a press office, please let them know about your upcoming paper now to help maximize its impact. If they'll be preparing press materials, please inform our press team within the next 48 hours. Your manuscript will remain under strict press embargo until 2 pm Eastern Time on the date of publication. For more information, please contact onepress@plos.org.

You will receive an invoice from PLOS for your publication fee after your manuscript has reached the completed accept phase. If you receive an email requesting payment before acceptance or for any other service, this may be a phishing scheme. Learn how to identify phishing emails and protect your accounts at https://explore.plos.org/phishing.

If we can help with anything else, please email us at customercare@plos.org.

Thank you for submitting your work to PLOS ONE and supporting open access.

Kind regards,

PLOS ONE Editorial Office Staff

on behalf of

Dr. Yury E Khudyakov

Academic Editor

PLOS One
